# Electrocardiogram Delineation in a Wistar Rat Experimental Model

**DOI:** 10.1155/2018/2185378

**Published:** 2018-02-08

**Authors:** Pedro David Arini, Sergio Liberczuk, Javier Gustavo Mendieta, Martín Santa María, Guillermo Claudio Bertrán

**Affiliations:** ^1^Instituto Argentino de Matemática “Alberto P. Calderón”, CONICET, Buenos Aires, Argentina; ^2^Instituto de Ingeniería Biomédica, Universidad de Buenos Aires, Buenos Aires, Argentina; ^3^Instituto de Ingeniería y Agronomía, Universidad Nacional Arturo Jauretche (UNAJ), Buenos Aires, Argentina; ^4^VECCSA-CardioVex, Buenos Aires, Argentina; ^5^Instituto de Investigaciones Médicas “Dr. Alfredo Lanari”, Buenos Aires, Argentina

## Abstract

**Background and Objectives:**

The extensive use of electrocardiogram (ECG) recordings during experimental protocols using small rodents requires an automatic delineation technique in the ECG with high performance. It has been shown that the wavelet transform (WT) based ECG delineator is a suitable tool to delineate electrocardiographic waveforms. The aim of this work is to implement and evaluate the ECG waves delineation in Wistar rats applying WT. We also describe the ECG signal of the Wistar rats giving the characteristics of its spectrum among other useful information.

**Methods:**

We evaluated a delineator based on WT in a Wistar rat electrocardiograms database which was annotated manually by experienced observers.

**Results:**

The delineation showed an “overall performance” such as sensitivity and a positive predictive value of 99.2% and 83.9% for P-wave, 100% and 99.9% for QRS complex, and 100% and 99.8% for T-wave, respectively. We also compared temporal analysis based ECG delineator with the WT based ECG delineator in RR interval, QRS duration, QT interval, and T-wave peak-to-end duration. The results showed that WT outperforms the temporal delineation technique in all parameters analyzed.

**Conclusions:**

Finally, we propose a WT based ECG delineator as a methodology to implement in a wide diversity of experimental ECG analyses using Wistar rats.

## 1. Introduction

Electrocardiograms (ECGs) are broadly used for diagnosing many cardiac pathologies as well as for researching on experimental animals. Since physiological information is present in ECG intervals and amplitudes, it is essential to develop delineation algorithms to obtain automatic characteristic wave peaks and boundary ECG points. Indeed, the performance of the ECG analysis depends on the accuracy of the technique employed in the electrocardiographic signal delineation. Moreover, ECG data emerging from long-term recordings, for example, Holter in humans or experimental protocols in animals, demand automatic ECG delineation with a good performance. ECG signal presents patterns with different frequency contents that repeat cyclically beat-to-beat. Typically, in the ECG delineation process, the algorithm firstly detects the QRS complex [1], which is the most pronounced wave of the heartbeat. Lastly, the delineator locates the onset, the peak, and the end of the P-wave, the QRS complex, and the T-wave.

There are several completely automatic methods for the delineation of ECG waveforms used in the literature, which are based on the phasor transform [2], mathematical models [3, 4], Hilbert transform [5], correlation analysis [6], template matching [7], *K*-nearest neighbor [8], artificial neural networks [9–11], hidden Markov model, hybrid hidden Markov models combined with the wavelet transform [12, 13], wavelet transform (WT) [14, 15], genetic algorithms, fuzzy logic methods, support vector machines, self-organizing maps, and Bayesian techniques [16], with each approach exhibiting its own advantages and disadvantages. Although various techniques have been considered, most of the studies presented in the literature use the wavelet transform technique, and researchers claim that this is the best method for extracting features from the ECG signal [11, 17, 18]. Moreover, analyzing several techniques mentioned previously [2–8], it can be observed that the WT technique presents the best results in terms of robustness and noise immunity [14]. This is because the WT method allows information extraction from both frequency and time domains, different from what is usually achieved by the traditional Fourier transform which permits the analysis of only the frequency domain. This way, the ECG can be described at different scales of temporal and frequency resolution, and thus high frequency waves (such as the QRS complex) can be distinguished from low frequency waves (such as P- and T-waves). Moreover, the WT can be easily implemented with a cascade of Finite-Impulse Response (FIR) filters [19]. In summary, the wavelet transform based ECG delineator (WT_D_) is a suitable tool to detect waveforms (such as mono- or biphasic P-wave, QRS complex, and mono- or biphasic T-wave) and is useful in different applications. Numerous methods, based on the wavelet transform ECG delineator technique proposed by Martínez et al., have been proposed [14–24].

As part of several research topics, a wide diversity of works utilized ECG analysis in small rodents. The rat and mouse models for research offer advantages with respect to larger animals such as lower cost, less physiological variability, and the possibility of using transgenic models. Normann et al. [25] analyzed ECG changes in rats during myocardial necrosis and infarction, both cases induced experimentally. Sgoifo et al. [26] showed ECG responses to different acute stressors in healthy rats, computing the heart rate variability (HRV) scores. Previously, they have implemented a software package developed to measure RR intervals and to interactively detect rhythm disturbances [27]. Also, telemetry systems have been widely used in rat experiments to analyze the relationship between ECG characteristics and behavioral responses during and after environmental challenges [28, 29]. Kuwahara et al. studied power spectral analysis of HRV in rats to assess autonomic nervous activity [30]. They developed an offline ECG analysis in order to detect R-waves and calculate the HRV. On the other hand, one investigation revealed that the stability of HRV estimators, for both time and frequency domain analysis in unrestrained rats, was highly dependent on the average heart rate (related to physical rat activity) as well as the length of the ECG records [31]. Moreover, Opitz et al. showed evidence that reperfusion can improve arrhythmia-related mortality by the prevention of ventricular fibrillation episodes [32]. They analyzed ECG offline in a semiautomatic fashion, as previously reported [33]. A recent work, making use of ECG biomarkers such as RR interval, QRS complex, and QT interval, showed that concomitant administration of trigonelline and sitagliptin produced a cardioprotective effect in contrast with monotherapy in diabetic rats [34]. Also, several toxicological studies showed the usefulness of ECG analysis in small rodents [35].

The aim of this work is to develop, validate, and provide a methodology for implementing WT_D_ for Wistar rat experimental protocols. We offer here a robust and noise-immune tool to delineate all the characteristic points of the ECG and not only the one corresponding to the R-wave. Complete delineation of the ECG allows evaluating not only the HRV but also the PR interval and P-wave duration in order to analyze atria activation and QRS complex with the aim of evaluating ventricular depolarization and QT interval for measuring ventricular depolarization and repolarization jointly and T-wave peak-to-end interval to quantify transmural ventricular repolarization. Also, amplitudes of the P-, R-, S-, and T-waves are in general all altered in heart diseases.

In this sense, we have focused on the following: (1) study the ECG signal in Wistar rats and its corresponding power spectral density, (2) implement and evaluate WT_D_ in an experimental model widely used in research, specifically Wistar rats, and (3) make a comparison between two ECG delineation systems in Wistar rats, such as WT_D_ versus temporal analysis based ECG delineator (TA_D_). Finally, despite the existence of software that detects and analyzes ECG parameters for different species such as pigs, dogs, rabbits, guinea pigs, mice, and rats [34, 36–38], to our knowledge, WT_D_ applied to Wistar rats has not previously been described.

## 2. Materials and Methods

### 2.1. Wistar Rats ECG Database

In order to analyze the WT_D_ algorithm in small rodent models, a Wistar rat database (WR_DB_) was developed. This database was acquired at the Institute of Medical Research “Alfredo Lanari” that belongs to the University of Buenos Aires, Argentina. Its contains a group of 57 Wistar rats, 29 males and 28 females, which were included in the study. When the measurements were carried out, this group contained 30 adult animals (424.5 ± 54.5 g) which were above 4 months of age and 27 young animals (245.5 ± 43.5 g) which were below 1 month of age. All these animals were in sinus rhythm at the time of ECG recordings.

After anesthesia with ketamine (75 mg/kg) and Rompun (0,75 mg/kg xylazine) administered subcutaneously and once the animal had been stabilized, the ECG signals were continuously recorded for at least 15 minutes in I-II, V_1_, V_3_, and V_6_ leads.

The ECG was acquired using EcoSur S.A. equipment (Buenos Aires, Argentina), digitized at 12-bit resolution with a sampling frequency of 1 kHz. These recordings were stored on a computer hard disk with custom-made software for posterior analysis.

In this work, we have only analyzed the delineation technique using lead II of the ECG, because it has shown robust P-, R-, S-, and T-waves. Lead II can be achieved in the Wistar rat by placement of the negative electrode near the right shoulder and the positive electrode to the left of the xyphoid space, in the same way as the Einthoven triangle (right arm position in the negative electrode and left leg position in the positive electrode).

Analysis of the ECG variables was carried out manually by two experienced observers (Ob_#1_ and Ob_#2_) using a computer calibrated cursor. Each ECG recording (57 in total) was annotated for each expert in 20 beats (1140 beats annotated), that is, a total of 2280 cardiac beats in order to validate WT_D_.

This process required manual measurement of 9120 characteristic ECG points by each expert observer in rat ECG measurement, that is, a total of 18240 electrocardiographic marks. The background noise level was 20 microV. To verify the reproducibility of the measurement methodology, 500 randomly selected recordings were analyzed by a third experienced observer (Ob_#3_). Animals were cared for according to Argentina's National Drug, Food and Medical Technology Administration Standards (Regulation 6344/96) and the National Institutes of Health* Guide for the Care and Use of Laboratory Animals* (NIH pub. number 85-23, revised 1996).

### 2.2. Temporal and Spectral Characteristics of the Wistar Rat ECG Signal

It is important to highlight that Q-wave does not exist in lead II WR_ECG_ [35], and hence we assumed the QRS_on_ temporal position as the start of the ventricular depolarization process. We also observed some special electrocardiographic characteristics in WR_ECG_, for example, as short QT interval, the absence of Q-wave in most ECG leads (in lead II, it does not exist), and the lack of ST segment. So, due to the absence of the ST segment in WR_ECG_, we observed that the beginning of the T-wave was located in the same position as the end of the QRS complex.

Also, normal ventricular repolarization was characterized by a J-wave which was identified as the down-sloping portion of the ST segment and could be attributed to the heterogeneity of the ventricular wall, as was described by Antzelevitch and Fish [39], who explained this phenomenon by IKto-mediated current present in the epicardium, but not endocardium.

We calculated the power spectrum of WR_ECG_ signals using 300 beats from six animals. This diagram represented the QRS complex and P- and T-waves power spectrum separately (Figure 2(b)). The ECG lead II has been used in order to plot the ECG power spectrum in Wistar rats. As far as we know, no results exist regarding ECG spectrum in Wistar rats. While, in H_ECG_, frequency components are approximately between 0.05 and 40 Hz, the range of WR_ECG_ goes from 4-5 to 120 Hz, as we can observe in Figure 2.

The spectral characteristic of a normal P-wave in humans is usually low frequency, below 10–15 Hz. Instead, most of the energy of the P-wave in rats is in the band between 20 and 60 Hz (see Figure 2). The most important frequency content of the QRS complex in humans is approximately between 5 and 25 Hz, while in WR_ECG_, this energy is situated in the band between 30 and 80 Hz. Finally, the T-wave in humans is similar to a P-wave below 15 Hz approximately, but with higher amplitude than P-wave, while in Wistar rats the T-wave ranges from 5 to 80 Hz.

### 2.3. Wavelet Transform

The WT provides an alternative to the short time Fourier transform for the analysis of nonstationary signals, utilizing short analysis windows at high frequencies and long analysis windows at low frequencies. This can be thought of as the decomposition of a signal in a set of basis functions, obtained by means of dilation (*a*) and translation (*b*) of a single prototype function wavelet called *ψ*(*t*). The WT of a signal *x*(*t*) is defined as $W_{a}x\left(b\right)=\left(1/\sqrt{a}\right)\int_{-\infty }^{+\infty }x\left(t\right)\psi \left(\left(t-b\right)/a\right)dt$, with *a* > 0. As the scale factor* a* increases, the wavelet becomes wider, giving information about lower frequency components of the signal, and upside down. The parameters *a* and *b* can be discretized according to a dyadic grid, being *a* = 2^*k*^ and *b* = 2^*k*^*l*. The transform is then called* dyadic wavelet transform, * with basis functions *ψ*_*k*,*l*_(*t*) = 2^−*k*/2^*ψ*(2^−*k*^*t* − *l*) with *k*, *l* ∈ *Z*^+^.

All details of WT_D_ have been extensively described in [14]; therefore, discrete time implementation is not explained in this work. The frequency responses of the first five scales were computed and obtained in the same way as Martínez et al. did [14].

However, it is important to highlight that the ECG delineation algorithm performs the detection of all characteristic points of the ECG waves using a quadratic spline WT, which, at scale 2^*k*^, is proportional to the derivative of the filtered version of the input ECG signal with a smoothing function at the same scale 2^*k*^.

In this sense, the zero crossings of the WT correspond to the minimum or maximum of the smoothed ECG signal at different *k* scales, and the maximum absolute values of the WT are related to maximum slopes in the smoothed cardiac electric signal. The local maximum, minimum, and zero crossings were used at different scales (2^1^ to 2^5^) in order to identify the characteristic points of the ECG signals.

### 2.4. WT Based Delineation Technique

In this section, we explain the delineation algorithms applied to Wistar rat electrocardiograms (WR_ECG_).

Because a priori we did not know the WR_ECG_ spectrum characteristics, we took the greatest amount of information that we could get, using a sampling rate of 1 kHz. Our system has been based on [14, 15]. There, the sampling frequency of H_ECG_ signals was 250 Hz. So, as Martínez et al. indicated in [14], we would normally have to redesign the filters according to the new sampling frequency and rescale the filter banks. But as we have mentioned before, WR_ECG_ spectrum bandwidth changed with respect to H_ECG_ spectrum and this balanced the cutoff frequencies in the WT scales, so that we did not need to change filter bank cutoff frequencies as described below.

The WT scales were compressed almost four times when the sample rate increased in the same order, producing a relative increase in the cutoff frequencies of each filter. That is, each curve belonging to a filter or a WT scale remained fixed (in the absolute sense of the equivalent frequency response) but changed its cutoff frequency in a relative way due to the change produced in the sampling frequency.

On the other hand, the input signal changed its bandwidth as it has been mentioned before. The WR_ECG_ signal did not have the same spectrum as H_ECG_ signal and it changed its maximum frequency, which increased nearly 3 times from 40 Hz to 120 Hz, compensating this way its frequency situation regarding the filters or wavelets scales which remained constant (in absolute terms). The compensatory effect kept the entire signal spectrum covered by the same number of filters (or scales) designed previously for validation when the ECG human sampling rate was four times lower. Finally, the maximum frequency spectrum of the signal in WR_ECG_ (120 Hz) coincided approximately with the cutoff frequency of a particular filter bank or WT scale (*k* = 2).

#### 2.4.1. QRS-Complex Detection

**Figure 1:**
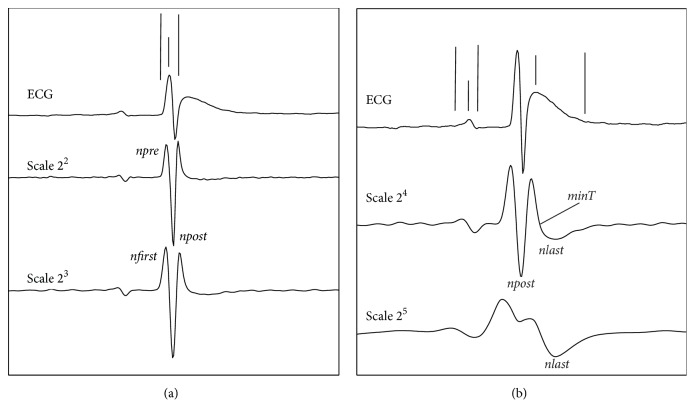
ECG beats delineation from WR_DB_. In (a), we can observe the QRS complex with its WT at scales 2^2^ and 2^3^ and the peak and QRS boundaries obtained by the algorithm proposed. The *npre* and *post* positions were located in scale 2^2^, while *nfirst* was located in scale 2^3^. In (b), we can see the P-wave and T-wave with their WT scales 2^4^ and 2^5^ and marks of peaks, onset, and end of characteristics ECG points. The *npost*, *last*, and *minT* positions were located in scale 2^4^, while *nlast* was located in scale 2^5^.

We built a vector containing maximum and minimum values above a certain threshold of scale 2 in WR_ECG_ and afterward a window of 15 ms in steps of 2 ms was shifted. Whenever a maximum following a lower maximum in that window is found, we call *npre* to the first maximum and *npost* to the second maximum. Then (in the modulus of scale 2) the minimum or lowest value between *npre* and *npost* was considered as the R-wave position in WR_ECG_ (see Figure 1(a)).

#### 2.4.2. QRS-Complex Delineation

The algorithm started from the R-wave previously detected, which had to be flanked by a pair of maximum moduli at |*W*_2^2^_*x*(*n*)| in WR_ECG_ that were called *npre* and *npost* (see Figure 1(a)).

From the position of maximum value *npost*, we have searched to the right within a window size of 15 ms until a maximum was found in |*W*_2^3^_*x*(*n*)| (see Figure 1(a)); this maximum corresponds to the end of the S-wave which also corresponds to the end of the QRS complex, so-called QRS_end_.

For the onset of the QRS complex, we search in |*W*_2^3^_*x*(*n*)| from the R-wave previously detected but to the left within a window of 15 ms long until a maximum was found; this point was called *n*_first_. Note that the Q-wave is not present in the rat signal, so from that position we searched a new point that conforms to a lower value with respect to the following threshold:

(1) $$\begin{array}{c} \xi QRS_{on}=0.35\left|\;W_{2^{3}}x\left(\;n_{first}\;\right)\;\right|. \end{array}$$

#### 2.4.3. T-Wave Delineation

For each R-wave position previously detected, we applied an analogous procedure to that applied in the QRS-complex boundaries detection. This way, we found the maximum after that corresponding R-wave, but this time in the scale |*W*_2^4^_*x*(*n*)| we call it *npost* (see Figure 1(b)). Note that, due to the absence of the ST segment in WR_ECG_, we observed that the beginning of the T-wave was located in the same position as the end of the QRS complex. Starting from this *npost*, we searched to the right for a maximum within a window size of 100 ms. Once this maximum was detected, we searched to the right in another window size of 100 ms to find a minimum; we call it *MinT* (see Figure 1(b)). The peak location of the T-wave had to be 2 ms before *MinT*. From the location of the aforementioned minimum, we searched the following maximum so-called *n*_last_. Finally, having found that point, *nlast*, we searched in a new window of 100 ms until we found a point in |*W*_2^5^_*x*(*n*)| (see Figure 1(b)) that was lower than the following threshold:

(2) $$\begin{array}{c} \xi T_{end}=0.45\left|\;W_{2^{5}}x\left(\;n_{last}\;\right)\;\right|. \end{array}$$

This value corresponds to the T-wave end position.

#### 2.4.4. P-Wave Delineation

The algorithm of P-wave delineation was analogous to the T-wave algorithm previously detailed (see Figure 1(b)). In this case, we searched to the left of the R-wave. The search windows to detect maximums and minimums were 40 ms of maximum length and the thresholds to detect the onset and offset of the P-wave were

(3) ξPon=0.6W24xnfirst,ξPend=0.9W24xnlast.

### 2.5. TA Based Delineation Technique in WR_ECG_

In order to extend the WR_DB_ analysis, we compared the WT_D_ technique versus the TA_D_ technique [40] using clinical ECG parameters such as RR interval, QRS duration, QT interval duration, and T-wave peak-to-end duration. Below, we briefly explained the TA_D_ technique as was described in the software LabChart 7 Pro v7.3.1, ADInstruments, Sydney, Australia [40].

The R-wave was identified as the most positive value in the neighborhood of the cardiac beat selected. QRS_on_ and QRS_end_ were determined by searches on each side of the R-wave for regions where the slope, computed as d*V*/d*t*, decreased to sufficiently low values. Moreover, the isoelectric level was computed as the median of all data values preceding QRS_on_. In this sense, P_peak_ was the point of the greatest absolute deviation from the isoelectric level, in an interval from pre-P-wave to just before QRS_on_. The P-wave was detected only if the peak exceeded a measure of uncertainty (the mean absolute deviation) in the isoelectric value. P_on_ was determined from a straight line fitted by least squares to points preceding P_peak_ (those points in the range 15–60% of the peak were used). The intersection of this line with the isoelectric level was P_on_; the search P_end_ was analogous to P_on_.

With respect to the T-wave delineation, searching was carried out for the first significant peak of either sign, starting from a point after QRS_end_. When T-wave was selected, the starting point was close to QRS_end_. If it was not selected, the starting point was further to the right. If a suitable peak was found, a straight line was fitted by least squares to the tail of the wave, over a data range 70–30% of T_peak_. Finally, T_end_ was found as the intersection of this line with the isoelectric level.

### 2.6. Validation and Evaluation of WT_D_

The evaluation in WR_DB_ was done between the reference ECG annotations provided by two experienced observers (gold standard) and WT_D_ implemented. We calculated the sensitivity as Se = Tp/(Tp + Fn) and the positive predictive value as *P*^+^ = Tp/(Tp + Fp). Tp (true positive) detection was considered when the ECG characteristic point was annotated and the algorithm detects its presence within a window < 25 ms in WR_DB_. Fn (false negative) detection was computed when the delineation algorithm located an ECG characteristic point which was not annotated by the expert. Lastly, a Fp (false positive) was calculated as the number of misdetections (the algorithm fails to locate the annotated characteristic ECG point within the abovementioned tolerance).

When there was no annotation of the ECG characteristic point, it was not possible to know whether the expert decided that there were no ECG characteristic points to mark or simply was not confident to mark it [14, 23]. Whatever the reason was, the *P*^+^ value was computed under the assumption that each absent mark meant that there was no waveform. Therefore, the computed *P*^+^ was interpreted as a lower limit so-called *P*_min_^+^ (see Table 1).

The mean value* “*Me*”* of the errors was computed as the temporal position difference between reference annotation and automatic delineation, and the average standard deviation* “*SD*”* of the error was computed by averaging the intra-ECG recording standard deviation (see Table 1).

### 2.7. Statistical Analysis in Comparison with Temporal Technique

Firstly, D'Agostino-Pearson's normality test was applied with the aim of quantifying the discrepancy between the ECG indices (RR interval, QRS duration, QT interval, and TPE duration) and ideal Gaussian distributions for each case. Secondly, because distribution variables were non-Gaussian, we applied the two-sided Mann–Whitney *U* test in unpaired samples and the Wilcoxon signed rank test in paired samples. Finally, in order to determine the statistical significance of ECG indices between temporal analysis and wavelet transform based ECG delineators, nonparametric tests were applied. When the *p* value was <0.05, differences were considered statistically significant.

## 3. Results

Figure 1 shows representative ECG beats obtained from WR_DB_. In (a), we can observe one beat with its QRS_on_, R_peak_, and QRS_end_ points detected, and their WT at scales 2^2^ and 2^3^ used for that purpose. Likewise, Figure 1(b) shows one beat with its P_on_, P_peak_, P_end_, T_peak_, and T_end_ characteristic points detected, and their WT at scales 2^4^ and 2^5^ utilized during the delineation process.

**Figure 2:**
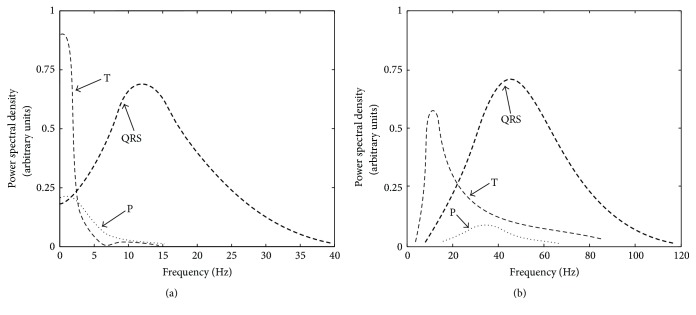
Representative power spectrum of ECG in human beings (a) and ECG in Wistar rats (b). The location of spectral contents in the P-wave, the QRS complex, and the T-wave can be observed.

Figure 2 presents the H_ECG_ (a) and WR_ECG_ (b) power spectrum characteristics. This diagram serves as a rough guide of where to locate the spectral components despite variations between beats of different leads, origins, and subjects.

The results obtained in WR_DB_ are given in Table 1; we observe the* “overall performance”* of WT_D_, which has been calculated as a mean representative value of Se and *P*^+^ in each electrocardiographic wave, that is, one unique value for P-wave, T-wave, and QRS-complex, respectively (see Table 1). Also, in this table, we show interobserver (Ob_#1_ versus Ob_#2_) annotation differences expressed as mean and SD, which were calculated over the same 1140 cardiac beats selected for manual measurement.

**Figure 3:**
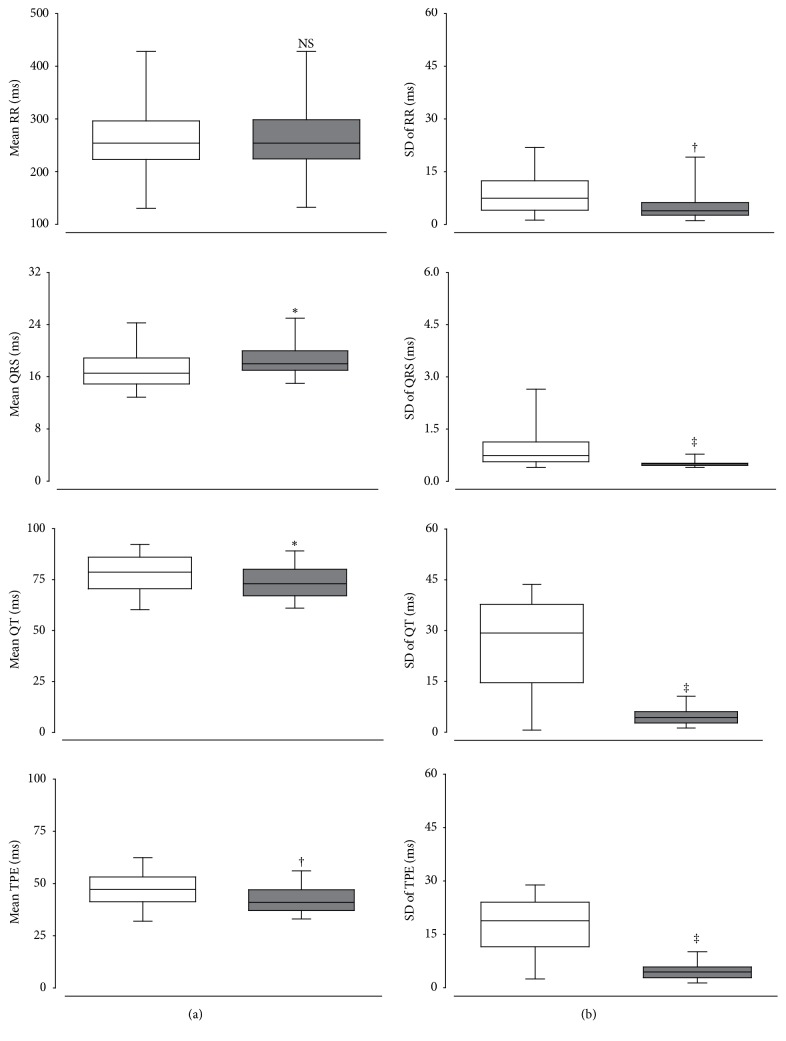
Box and whisker plots showing the mean (a) and standard deviation (b) values for different ECG parameters in Wistar rats. The ECG parameters measured were the RR interval (RR), QRS duration (QRS), QT interval duration (QT), and T-wave peak-to-end duration (TPE). The temporal analysis based ECG delineator (TA_D_) was represented with clear boxes and the wavelet transform based ECG delineator (WT_D_) was represented with dark boxes. ^*∗*^*p* < 0.05, ^†^*p* < 0.001, and ^‡^*p* < 0.0001 indicate statistically significant differences of ECG parameters between TA_D_ and WT_D_. NS: nonstatistically significant differences between both groups.

The purpose of Figure 3 is to compare two delineation techniques, namely, temporal analysis based ECG delineator (TA_D_) and wavelet transform based ECG delineator (WT_D_), for measuring ECG parameters with clinical utility, such as RR interval, QRS duration, QT interval duration, and T-wave peak-to-end duration. Box and whiskers plots of the mean value and the standard deviation of both WT_D_ (Section 2.4) and TA_D_ (Section 2.5) techniques are reported.

## 4. Discussion

The ECG waves delineation in Wistar rats provides fundamental features such as amplitude and duration of each cardiac beat. This information can be utilized in order to evaluate pathologies, behavior, and/or drug effects [35]. In this sense, it is necessary to achieve very good performance to detect characteristic waves, peaks, and boundaries of ECG points.

### 4.1. The WT_D_ Performance in Wistar Rats

We have implemented WT_D_ in signals obtained from WR_DB_, as we can see in Table 1. We observed very good sensitivity and positive predictive values with respect to both experienced observers (Ob_#1_ and Ob_#2_) in P-wave, QRS complex, T-wave delineation, and QRS detection. Taking into account the delineation of the onset, the peak, and the end, we observed that Se values were higher than 99% in the P-wave, equal to 100% in the QRS complex and T-wave, and likewise 100% in the QRS detection. Regarding the positive predictive value, it ranged from 82% up to 87% in the P-wave delineation and was approximately 100% in the QRS-complex and T-wave delineation. Also, in the QRS detection, it was approximately 100%. We did not observe statistically significant differences between the results presented by both observers (*p* = NS). In conclusion, we observed a high performance to delineate WR_ECG_ signals regardless of the observers, that is, Ob_#1_ or Ob_#2_. Also, in order to verify the reproducibility of the measurement methodology, 500 randomly selected ECG recordings were analyzed by a third observer (Ob_#3_). The interobserver differences between both Ob_#1_ versus Ob_#3_ and Ob_#2_ versus Ob_#3_ were not significant (NS).

The difference in performance between sensitivity and positive predicted value in the data is presented only in the detection of the P-wave, but not in the rest of the waves, and is due to different factors, such as the absence, low amplitude, M-shape, and/or biphasic morphology of the P-wave. This leads to having more false positives (Fp) than false negatives (Fn), which finally explains the difference in performance.

### 4.2. Comparison with Temporal Techniques

In order to analyze the delineation performance in WR_ECG_, we compared WT_D_ proposed in this work with the commercial software LabChart 7 Pro version 7.3.1 (ADInstruments, Sydney, Australia, [40]); the latter was based on  TA_D_ technique. Electrocardiographic parameters such as RR interval, QRS duration, QT interval duration, and T-wave peak-to-end duration were computed from both methods named above using frontal ECG lead II.

In Figure 3, we can observe box and whisker plots of the mean (a) and standard deviation (b) of the mean values of 57 Wistar rats (40 beats processed for each animal) for relevant ECG parameters.

When we compared the WT_D_ technique with the TA_D_ technique, we observed that our algorithm outperforms clearly the temporal technique when the standard deviation values were computed. Moreover, statistically significant differences between both delineation techniques can be observed.

### 4.3. ECG Variables in Normal and Anesthetized WRs

Recently, the ECG time intervals (registered in lead II) have been presented in unanesthetized WRs [41]. Representative values, the mean, and the range from the minimum to the maximum values included between square brackets were shown in [41] as seen below: HR 460 [228–600] bpm, P 13 [10–16] ms, PR 40 [33–50] ms, QRS 17 [12–26], and QT 66 [38–80] ms.

In order to compare our results with [41], we have computed the average values mean ± SD of the animals used in the present work, which are detailed below: HR 251 ± 22 bpm, P-wave 24.5 ± 2.0 ms, PR interval 54.7 ± 5.3 ms, QRS complex 17.9 ± 1.6 ms, and QT interval 83.3 ± 4.0 ms. It can be observed that the major discrepancy was in the P-wave definition. Moreover, representative amplitude values, mean, and range were reported in [41] as seen below: P 110 [20–200] uV, Q 0 uV, R 1060 [220–1500] uV, S 200 [0–500] uV, and T 150 [50–300] uV. Also, we have calculated the average values mean ± SD, which are presented below: P-wave 93.8 ± 40.6 uV, Q-wave 0 uV, R-wave 610.8 ± 140.1 uV, S-wave −385.2 ± 152.3 uV, and T-wave 163.8 ± 58.4 uV. The values obtained in this work do not present discrepancy with unanesthetized WRs presented in [41]. We emphasize that normal variants presented in [41] cannot be considered abnormal. Finally, it has been concluded that the values of the ECG parameters in anesthetized WRs did not change rhythm or morphology in any way or affected the results of the delineation.

## 5. Conclusions

There are a lot of experimental works which have shown the utility of ECG in small rodents in order to discern the mechanisms of action of drugs, infer disturbances of the cardiac rhythm and electrical conduction, detect the presence of ischemic injury, and analyze behavioral responses, among others. In spite of being a useful tool, it is not sufficient as a standalone methodology to describe abnormal electrophysiological mechanisms. However, when combined with other electrophysiological techniques that can only be carried out in animal models, the ECG analysis may be used to obtain a more complete understanding of the electrical cardiac mechanism. Also, it is important to highlight that replacement of printed ECG with digitized ECG, as well as delineation algorithms with high performance values, is collectively streamlining ECG assessments in experimental protocols.

In the present work, we have shown and validated the capability to perform QRS detection and location of ECG wave boundaries using WT_D_ in ECG Wistar rats. Also, the methodology implemented has provided reliable and accurate delineation in the ECG in comparison with the TA technique delineation. Finally, due to the importance of having a high performance in the ECG delineated stage, we propose the use of WT_D_ during experimental protocols which use Wistar rats.

## Figures and Tables

**Table 1:** Performance of wavelet transform based ECG delineator applied to Wistar rats ECG database. We can observe the results of sensitivity and positive predictive value of this work in comparison with both experienced observers (Ob_#1_ and Ob_#2_). The analysis of the ECG variables was carried out manually by two experienced observers using a computer calibrated cursor. Also, interobserver (Ob_#1_ versus Ob_#2_) annotation differences mean and variability are presented. Moreover, delineation of WR_DB_ showed an overall performance that was computed as a mean value between the characteristic points for each electrocardiographic wave, such as Se and *P*^+^ of 99.2% and 83.9% for P-wave, 100% and 99.9% for QRS complex, and 100% and 99.8% for T-wave, respectively.

	Param.	P_on_	P_peak_	P_end_	QRS_on_	R_peak_	QRS_end_	T_peak_	T_end_
This work versus Ob_#1_	Se (%)	99.3	99.3	99.3	100	100	100	100	100
	*P* ^+^ (%)	82.1	82.1	82.1	100	100	100	100	100
	Me ± SD (ms)	2.59 ± 3.99	0.33 ± 3.93	0.47 ± 3.86	0.66 ± 1.72	−0.04 ± 1.4	0.03 ± 2.15	1.72 ± 3.05	2.40 ± 11

This work versus Ob_#2_	Se (%)	99.1	99.1	99.07	100	100	100	100	100
	*P* ^+^ (%)	86.65	87.06	87.27	99.8	100	99.6	99	100
	Me ± SD (ms)	3.4 ± 5.58	0.17 ± 4.81	0.21 ± 4.61	0.03 ± 1.73	−0.21 ± 0.63	0.39 ± 1.86	1.59 ± 2.9	−1.75 ± 9.2

Me ± SD (Ob_#1_ versus Ob_#2_) (ms)		0.72 ± 2.38	−0.2 ± 1.31	−0.38 ± 1.93	−0.63 ± 2.16	−0.17 ± 1.45	0.36 ± 1.87	−0.14 ± 2.14	−4.1 ± 9.58

Overall	Se (%)	99.2			100			100	
Performance	*P* ^+^ (%)	83.9			99.9			99.8	

## Acronyms

ECG: Electrocardiogram
H_ECG_: Human being electrocardiograms
*P*^+^: Positive predictive value
Se: Sensitivity
TA: Temporal analysis
TA_D_: Temporal analysis based ECG delineator
WR_DB_: Wistar rats database
WR_ECG_: Wistar rats electrocardiogram
WT: Wavelet transform
WT_D_: Wavelet transform based ECG delineator.
